# Examining the Effect of Charged Lipids on Mitochondrial Outer Membrane Dynamics Using Atomistic Simulations

**DOI:** 10.3390/biom12020183

**Published:** 2022-01-22

**Authors:** Aline A. Oliveira, Tomasz Róg, Albérico B. F. da Silva, Rommie E. Amaro, Mark S. Johnson, Pekka A. Postila

**Affiliations:** 1 Department of Chemistry and Biochemistry, University of California San Diego, San Diego, CA 92093-0340, USA; line_quimica@hotmail.com (A.A.O.); ramaro@ucsd.edu (R.E.A.); 2 Instituto de Química de São Carlos, Universidade de São Paulo, CP 780, São Carlos 13560-970, Brazil; alberico@iqsc.usp.br; 3 Department of Physics, University of Helsinki, P.O. Box 64, FI-00014 Helsinki, Finland; tomasz.rog@helsinki.fi; 4 Structural Bioinformatics Laboratory, Biochemistry, Faculty of Science and Engineering, Åbo Akademi University, 20520 Turku, Finland; mark.s.johnson@abo.fi; 5 InFLAMES Research Flagship Center, Åbo Akademi University, 20520 Turku, Finland; 6 Institute of Biomedicine, Kiinamyllynkatu 10, Integrative Physiology and Pharmacy, University of Turku, FI-20520 Turku, Finland; 7 Aurlide Ltd., FI-21420 Lieto, Finland; 8 InFLAMES Research Flagship Center, University of Turku, FI-20520 Turku, Finland

**Keywords:** outer mitochondrial membrane (OMM), molecular dynamics (MD), negatively charged lipids, phosphatidylinositol (PI), phosphatidylserine (PS), membrane lipid composition (MLC), schizophrenia, monoamine oxidase B (MAO-B)

## Abstract

The outer mitochondrial membrane (OMM) is involved in multiple cellular functions such as apoptosis, inflammation and signaling via its membrane-associated and -embedded proteins. Despite the central role of the OMM in these vital phenomena, the structure and dynamics of the membrane have regularly been investigated in silico using simple two-component models. Accordingly, the aim was to generate the realistic multi-component model of the OMM and inspect its properties using atomistic molecular dynamics (MD) simulations. All major lipid components, phosphatidylinositol (PI), phosphatidylcholine (PC), phosphatidylethanolamine (PE), and phosphatidylserine (PS), were included in the probed OMM models. Because increased levels of anionic PS lipids have potential effects on schizophrenia and, more specifically, on monoamine oxidase B enzyme activity, the effect of varying the PS concentration was explored. The MD simulations indicate that the complex membrane lipid composition (MLC) behavior is notably different from the two-component PC-PE model. The MLC changes caused relatively minor effects on the membrane structural properties such as membrane thickness or area per lipid; however, notable effects could be seen with the dynamical parameters at the water-membrane interface. Increase of PS levels appears to slow down lateral diffusion of all lipids and, in general, the presence of anionic lipids reduced hydration and slowed down the PE headgroup rotation. In addition, sodium ions could neutralize the membrane surface, when PI was the main anionic component; however, a similar effect was not seen for high PS levels. Based on these results, it is advisable for future studies on the OMM and its protein or ligand partners, especially when wanting to replicate the correct properties on the water-membrane interface, to use models that are sufficiently complex, containing anionic lipid types, PI in particular.

## 1. Introduction

Membrane lipid composition (MLC), especially anionic lipids, can affect lipid bilayer and membrane protein dynamics, structure and function [1,2,3,4]. The MLC has been shown to affect small, drug-like molecules partitioning and translocation through a membrane [5]. Moreover, MLC differences have also been suggested to affect receptor entry processes of drugs and neurotransmitters [6,7,8]. Accordingly, it is vital to determine the biologically relevant MLCs and then utilize these lipidomics data to reveal the exact roles of the specific lipid components and contribution to properties of a membrane. Such studies have been performed for, e.g., exosomes [9,10], yeast endoplasmic reticulum, plasma membrane, and trans-Golgi network [11], and adipose tissue [12].

Prior experimental studies have indicated that specific lipid composition changes of the mitochondrial outer membrane (OMM) can affect the function of membrane proteins such as monoamine oxidase B (MAO-B) [13,14]. MAO-B is a monotopic membrane protein that degrades, e.g., benzylamine and phenylamine, but, most notably, monoamine neurotransmitters such as dopamine [15]. Thus, the OMM composition directly or indirectly affects the regulation of the synaptic dopamine level and, thus, is crucial for treating neurological disorders such as Parkinson’s disease and depression [16]. Despite the apparent importance of OMM for a multitude of cellular functions such as apoptosis [17], inflammation [18,19], and monoamine degradation [20], the tendency has been to oversimplify the OMM composition in prior in silico studies, e.g., [21,22,23]. It is prudent to represent or inspect the OMM with the most accurate and current lipid compositions thus updating the situation regarding the roles of different lipid species and ensuring that future studies on membrane proteins involving the OMM are performed with the most accurate MLC possible. Notably, a major anionic component, phosphatidylinositol (PI; 13% of OMM mass; [24,25]), has been excluded from prior simulation studies concentrating on the OMM [21,22,23]. Because the necessary parameters are now available for the CHARMM36 force field [26], a representative of these anionic lipids can now be included in the studied model systems.

This in silico study aimed to explore the effect of a complex MLC for the OMM using atomistic molecular dynamics (MD) simulations (Models #1–5; Table 1). Firstly, we wanted to examine how the simple two-component model (Model #5; Table 1) performs in comparison to a model housing all the major lipids of OMM in correct ratios (Model #2; Table 1). Secondly, we also wanted to see how the varying of PS concentration affected the membrane structure and dynamics (Models #1–4). The increased levels of this anionic lipid species have been linked to schizophrenia [14] and, more specifically, to the inactivation of the MAO-B enzyme [13]. Thirdly, the idea was to set the foundation for pursuing more simulation work using these membrane models in the future with small-molecules (e.g., dopamine) and membrane proteins residing at the OMM (e.g., MOA-B). The membrane environment affects neurotransmitter dynamics throughout their life cycle, which also includes their removal from the synaptic cleft, intracellular synthesis and eventual degradation (e.g., dopamine + MAO-B) [7].

The results indicate that the four-component OMM models produce notably different results than the previously applied two-component models containing phosphatidylcholine (PC) and phosphatidylethanolamine (PE) [27]. There are changes in the bilayer thickness, area per lipid values, lateral diffusion rates, membrane surface charge, and the amount of membrane-ion contacts directly attributed to the lipid composition. The results suggest that the overall effect of the accurate OMM lipid composition on the water-membrane interface should not be ignored in future studies, when for example studying the membrane protein conformation and dynamics.

In short, based on the simulations, we present an OMM model (Model #2; Table 1) with realistic lipid composition, which is ready for various membrane protein simulations. These equilibrated membrane model coordinates are made freely available (see the Supplementary File simulated_systems.zip).

## 2. Materials and Methods

### 2.1. Outer Mitochondrial Membrane Models

In total, five models of the outer mitochondrial membrane (OMM) with varying membrane lipid composition (MLC; Table 1) were generated using the CHARMM-GUI (http://www.charmm-gui.org/ (accessed on 1 June 2015)) [28,29] and the CHARMM36 lipid force field [30]. All major lipid components, including phosphatidylinositol (PI), phosphatidylcholine (PC), phosphatidylethanolamine (PE) and phosphatidylserine (PS), were included in the OMM models in different ratios (Table 1). The size of each membrane model is relatively big (784 lipids; ~338,000 atoms), because they are meant to be large enough for future membrane protein simulations as well. The systems were solvated using TIP3P (transferable intermolecular potential with 3 points) water molecules. Additionally, 150 mM Na^+^/Cl^−^ counter ions were added to the systems to neutralize the total charge and assure physiologically relevant ionic content.

### 2.2. Molecular Dynamics Simulations

The molecular dynamics (MD) simulations were performed using NAMD2.9 [31] and the default input settings were provided by the CHARMM-GUI [28,32]. Firstly, each OMM model was energy minimized (10,000 steps) and equilibrated in two phases for 50 ps using canonical ensemble (NVT) simulations at 310 K. Secondly, 625 ps isothermal-isobaric (NPT) ensemble simulations were performed in four phases. Planar restraints that hold the lipid head/tail groups in place along the Z-axis and dihedral restraints that hold the chirality of lipid headgroups and double bonds were gradually decreased during the equilibration steps [33]. The 300 ns free NPT production simulations were performed using a pressure of 1 atm. Electrostatic interactions were treated using the Particle Mesh Ewald (PME) method [34,35] and a time step of 2 fs was used in the simulations. Total energy, temperature, pressure, periodic box dimensions and membrane thickness or partial density profiles of the simulations indicated that the systems were stable and remained close to the set reference values, and are given in the Supplementary Materials (Figures S1–S5). The MD simulation trajectories were also visually inspected for any potential stability issues such as excessive membrane undulations or bending.

### 2.3. Analysis and Figure Preparation

The analysis of the simulations was performed using the tools included in GROMACS [36]. The first 20 ns of the production simulations was excluded from the analysis. VMD 1.9.3 was used to draw the membrane models in 3D [37].

## 3. Results

### 3.1. Phosphatidylinositol Increases the Surface Area per Lipid

The area per lipid (APL) values of the five probed membrane models (Models #1–#5 in Table 1) did not differ markedly from each other (Table 2). In fact, the APL averages ranged from 61.2 Å to 62.1 Å (Table 2) with the smallest APL value observed for the neutral Model #5, which is composed of POPC and POPE lipids. The increase of APL in the membrane models containing negatively charged lipid species is the likely cause for the reduction in the membrane surface charge density (Table 2). The increased APL values for membrane models containing a high concentration of PI(3,4)P2 (Model #1 and Model #2 in Table 1) is explained by the large size of its anionic lipid headgroup.

There are no direct experimental data to compare the MD simulation results against with these particular membrane lipid compositions (MLCs). Nevertheless, there exist reliable APL data on the two main lipid components of the membrane models: area per POPC and POPE are 0.643 [38] and 0.566 nm^2^, respectively, at 303 K [39]. Thus, assuming ideal mixing of POPC and POPE, the APL should be close to 0.610 nm^2^—a result that is almost matched by the equivalent two-component membrane at 310 K (Model #5 in Table 2).

### 3.2. Phosphatidylserine Increases the Bilayer Thickness

**Figure 1:**
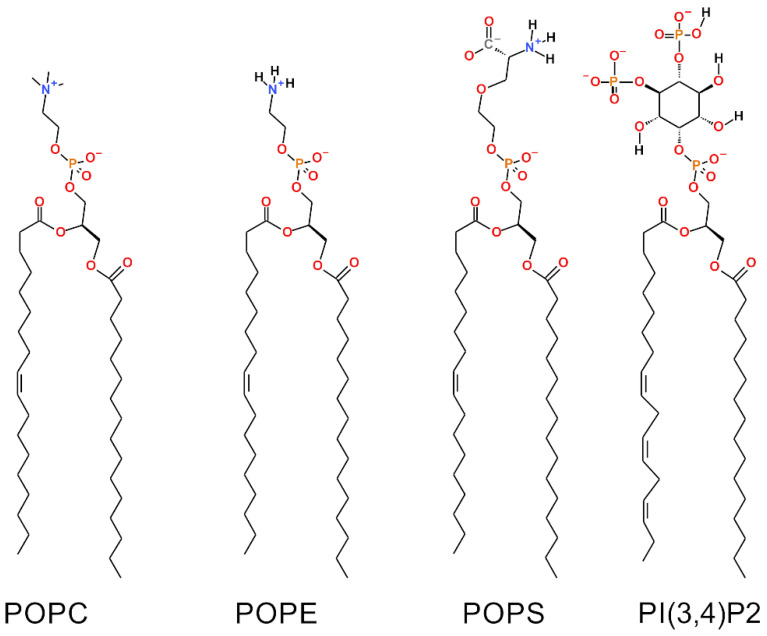
Chemical structures of the lipid molecules included in the outer mitochondrial membrane models. The 2D representations of: phosphatidylcholine (POPC), phosphatidylethanolamine (POPE), phosphatidylserine (POPS) and phosphatidylinositol (PI(3,4)P2).

The bilayer thickness values of the membrane models (Table 2) were calculated based on the distances between the lipids’ phosphate groups (Figure 1; Figure S5) in the inner and outer leaflets from the density profiles. The thickness did not markedly differ from one membrane model to the next as the results ranged from 4.06 to 4.19 (Table 2). However, interestingly, the addition of negatively charged lipid components consistently led to small decreases in the membrane thickness. Although the individual effects of POPS and PI(3,4)P2 were not studied exhaustively for comparison, the latter anionic lipid clearly had a bigger effect on the membrane thickness.

### 3.3. Negatively Charged Lipids Lower the Order Parameter

**Figure 2:**
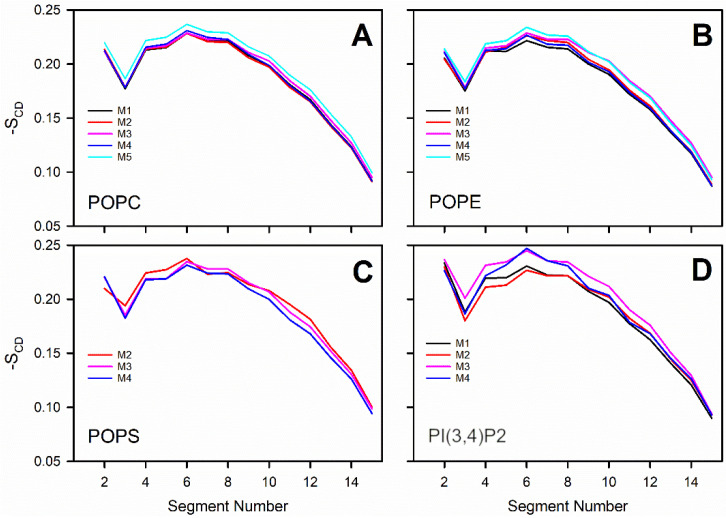
Molecular order parameter -S_CD_: comparison of the models for each lipid species: (**A**) POPC, (**B**) POPE, (**C**) POPS, and (**D**) PI(3,4)P2 (Figure 1). The membrane models (Models #1–#5 or M1–M5; Table 1) are shown with distinct colored lines.

**Figure 3:**
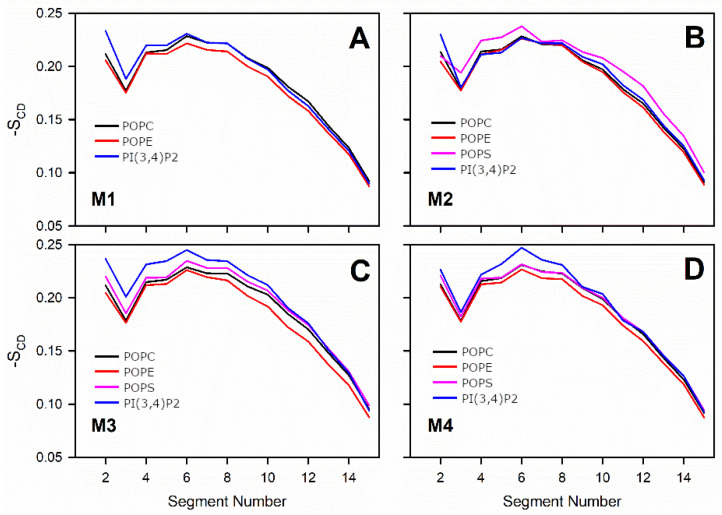
Molecular order parameter -S_CD_: comparison of the lipid species in each model: (**A**) Model #1, (**B**) Model #2, (**C**) Model #3, and (**D**) Model #4 (or M1–M4; Table 1). The different lipid species are shown with distinct colored lines (Figure 1).

In membranes, the phase transitions can be described by the molecular deuterium order parameter (S_CD_) that is defined as:

(1) $$S_{\mathrm{CD}}=\left\langle \frac{3}{2}\left({cos}^{2}\theta_{i}\right)-\frac{1}{2}\right\rangle$$

where *θ_i_* is the angle between the C-D bond (C-H in simulations) of the *i*-th carbon atom and the bilayer normal [40]. The angle brackets denote averaging over time and over appropriate C-D bonds in the bilayer. S_CD_ parameter profiles along the saturated tail (sn1) of each OMM model or specific lipid species are shown in Figure 2 and Figure 3. For POPC and POPE, the order parameter was decreased somewhat with all membrane models containing negatively charged lipids in comparison to the neutral POPC-POPE bilayer (Models #1–4 vs. Model #5 in Figure 2). The acyl tails of PI(3,4)P2 (Figure 1) were more ordered in Models #3 and #4, which contained larger amounts of POPS lipids than the other models. These results are not surprising since the surface area, membrane thickness, and order parameter are geometrically interdependent [41].

### 3.4. Phosphatidylserine Reduces the Lateral Diffusion

In order to evaluate the effect of MLC on translational motion of the lipids, the mean squared displacement (MSD) of the center of mass of the lipids molecules in the bilayer plane was calculated and fitted to the diffusion coefficient (Table 3; Figure S6) according to the equations:

(2) $$MSD\left(\tau \right)={\langle \left|r\left(t_{0}\right)-r\left(t_{0}+\tau \right){|}^{2}\right\rangle }_{t_{0}}$$

where *r* is the position of the molecule, *t*_0_ and *t* are the initial and lap times (specific time intervals), and $\langle ...\rangle_{t_{0}}$ denotes an averaging over different initial times within a simulation run and over all molecules in the system.

(3) $$D=\underset{n\to \infty }{\lim}\frac{MSD}{2N_{f}t}$$

where *N_f_* is the number of degrees of translational freedom for the molecule (2 for lateral diffusion).

The highest lipid diffusion coefficients were seen in Models #1 and #5 (Table 3), which lack POPS altogether (Table 1). The addition of POPS into the membrane model induced a decrease of the lateral diffusion rate for all lipid types in Models #2–#4. Additionally, in these three models the charged lipids are characterized by slower diffusion rates. In contrast, this effect was not observed for Model #1, in which the PI(3,4)P2 diffusion rate remained comparable to POPC and POPE. Notably, as the relatively large error estimate values suggest (Table 3), these results should be regarded as qualitative only, as considerably longer simulations and larger models are needed with the used simulation set-up for the quantitative assessment of the lipid diffusion coefficients [42].

### 3.5. Charged Lipids Reduce the Rotational Diffusion Rate of the POPE Headgroup

**Figure 4:**
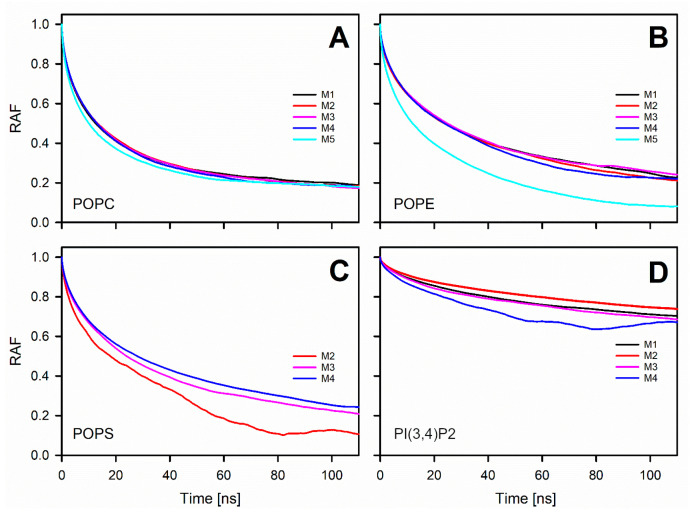
Rotational autocorrelation functions of the lipid headgroups in membrane models for each lipid species: (**A**) POPC, (**B**) POPE, (**C**) POPS, and (**D**) PI(3,4)P2. (Figure 1) The membrane models (Models #1–#5 or M1–M5, Table 1) are shown with distinct colored lines.

As a means to describe the rotational motion of the lipid headgroup, we considered the rotational autocorrelation functions (RAF) of the vector between phosphate and nitrogen atoms for POPC, POPE, and POPS, and atoms C2 and C5 in the inositol ring of PI(3,4)P2 (Figure 1):

(4) $$C_{l}\left(\tau \right)=\langle P_{l}\left(n\left(t_{0}\right)\cdot n\left(t_{0}+\tau \right)\right)\rangle_{t_{0}}$$

where *P_l_* is the *l*-th Legendre polynomial, *n* is a fixed unit vector in the molecule, *t*_0_ and *τ* are the initial and lap times, respectively, and ${\langle ...\rangle }_{t_{0}}$ denotes an averaging over different initial times within a simulation run and over all molecules in the system. In this study, Legendre polynomial *P_l_*(cos*θ*) = cos*θ* was first considered. Figure 4 clearly shows that rotation of the POPC headgroup is only slightly affected by the presence of the charged lipids; meanwhile, the rotation of the POPE headgroup is markedly reduced or slowed down whenever the membrane model contained charged lipid species. Surprisingly, specific charged lipid compositions did not result in large differences among the membrane models. Rotation of the POPS headgroup is also affected by the MLC: it slows down in the bilayers with a relatively high concentration of POPS. No notable differences were observed in the lipid headgroups’ orientations measured as the angle between the PN (Phosphate-Nitrogen) vector and bilayer normal (data not shown). 

### 3.6. Interactions at the Water-Membrane Interface

To describe interactions at the water-membrane interface, the numbers of hydrogen bonds (or H-bonds) and charge pairs were calculated. A H-bond was defined as having (1) a distance between the hydrogen bond donor (D) and acceptor (A) less than 0.35 nm; and (2) the angle between the vector D-A and the D-H covalent bond had to be less than 35°. The charge pairs (or ionic bonds) between the positively charged methyl groups (Me) of choline and negatively charged oxygen atoms (Figure 1) were evaluated based on the Me-O distance, which had to be less than 0.4 nm.

Firstly, the number of H-bonds was calculated between the lipids and water molecules (Table 4). The POPC hydration was hardly affected by the presence of charged lipids as it increases only ~1% with those membrane models containing charged lipids in comparison to the neutral POPC-POPE bilayer (Models #1–#4 vs. Model #5; Table 4). In contrast, the POPE hydration decreased in response to the presence of charged lipids, the largest decrease of POPE hydration (6% drop) was observed between Models #3 and #5. The POPS hydration decreased, when the surface charge density or POPS concentration increased, resulting in a hydration difference of 5% between Models #2 and #4. With PI(3,4)P2, no clear trend was observed; however, the difference in the PI(3,4)P2 hydration between Models #2 and #4 was ~7%.

Secondly, the number of H-bonds and charge pairs formed among the lipid molecules in the models was determined. In Table 5, the number of expected H-bonds assuming equal affinity of the lipid species towards each other is also provided (in square brackets), thus, this ideal H-bond number depends only on the lipid concentration (Table 1). The primary finding of this inter-lipid analysis was the lower frequency of H-bonds between POPC and all the remaining lipids (Table 5): the observed number of H-bonds was in most cases less than half of the expected H-bond count. This effect was the strongest for PI(3,4)P2, followed by POPS, and POPE. POPC, instead, forms a substantial number of charge pairs with all three lipid types. In particular, PI(3,4)P2 participates in a high number of charge pairs, about 4 per PI(3,4)P2 molecule. POPS participate in a smaller number of charge pairs, 1.3–1.8 per POPS molecule. Finally, POPE forms the lowest number of charge pairs, about 1 per POPE molecule. Considering H-bonds where POPE is a hydrogen donor, H-bonds with PI(3,4)P2 are 2–3 times more frequent than what was expected if everything was considered equal.

Thirdly, we calculated the number of sodium ions (Na^+^) interacting with lipids using the minimum distance of 0.325 nm between Na^+^ and lipids oxygen atoms as a criterion (Table 1 and Table 6). Data shown in Table 6 indicate that PI(3,4)P2 attracts more Na^+^ than POPS and the neutral lipids do not interact very much with Na^+^. This difference in Na^+^ adherence leads to an overall charge difference at the water-membrane interface; i.e., those membrane models containing large amounts of PI(3,4)P2 have a relatively neutral interface in comparison to models in which POPS was the main anionic component (Table 1). Moreover, a larger number of lipid oxygen atoms coordinates Na^+^ ions in the bilayers with higher levels of PI(3,4)P2.

## 4. Discussion

**Figure 5:**
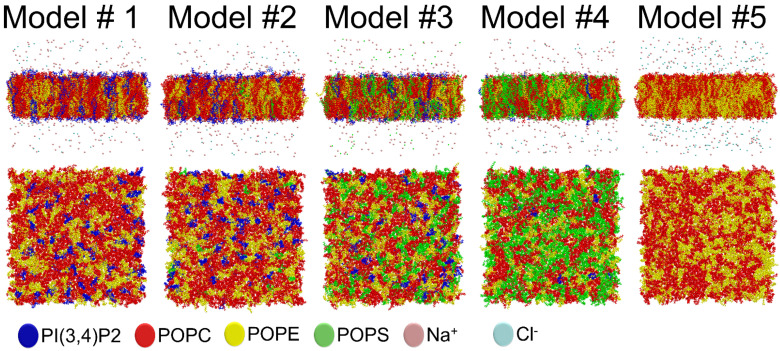
The 3D structures of the outer mitochondrial membrane models after 300 ns simulation. On top are shown lipid bilayers or membrane models (Table 1) from the side view. Below are shown the lipid headgroup region of one membrane leaflet from the top view. The different lipid species and ions are shown as stick models with distinct colors. Solvent is omitted for clarity.

Prior in silico studies focusing on properties of the outer mitochondrial membrane (OMM) have been conducted using relatively simple single- or two-component membrane models [28,43,44]. However, due to ongoing advancements in lipid force field development [43,45,46], it is becoming increasingly possible to study more realistic and complex membrane lipid compositions (MLCs) than ever before by using atomistic molecular dynamics (MD) simulations. Here, the MD simulations were performed using models with 2–4-lipid components (Table 1; Figure 1) and, thus, the interplay and combined effect of all major OMM lipid types [24,25] could be established (Figure 2, Figure 3, Figure 4 and Figure 5; Table 2, Table 3, Table 4 and Table 5).

Notably, the effects of two charged lipid types, PI(3,4)P2 (1-palmitoyl-2-linolenoyl-inositol-(4,5)-bisphosphate (PIP2) with protonation on P4; Figure 1) and POPS (phosphatidylserine; Figure 1), were probed by varying their concentration among the five OMM models (Models #1–#5; Table 1; Figure 5). Overall, the results show that the structural properties such as membrane thickness, area per lipid, and S_CD_ order parameter were not primarily affected by the MLC changes within the studied membrane models. These basic metrics were close to the equivalent numbers found for neutral POPC-POPE membrane in the simulations (Model #5 in Table 1 and Figure 5) and experiments [38,39]. This behavior was to be expected based on prior simulation studies, which similarly have shown that charged lipids at moderate physiological concentrations induce only minor effects on the structural properties of membranes [23,44,47] or even phase behavior [48]. However, the effect of charged lipids may be modulated by other factors, e.g., phosphatidic acid significantly affects the ordering of the bilayer composed of saturated lipids while its effect on unsaturated lipids is tiny [49].

The detailed analysis of the trajectories from the MD simulations indicated that the changes in the charged lipid levels of the OMM models cause substantial effects on the dynamical parameters. The results suggest that increasing the POPS levels in the OMM models slows down the lateral diffusion of all lipids forming the bilayer. The second observation is that the headgroup rotation of POPE was slowed down in the OMM models containing charged lipid species without POPS or PI(3,4)P2-specific interactions. The rotation of the POPS headgroup was also slowed down, but only in response to high POPS levels.

The water-membrane interface, where the OMM simulations are likely to be more accurate than on the above-mentioned diffusion metrics, is expected to be affected by any changes to the lipid headgroup [50,51,52,53]; thus it is not surprising that there are indeed notable differences between studied models. In general, the POPE hydration was reduced when anionic lipids were included (Table 4). The surface charge density or POPS concentration increase generated a hydration difference of 5% for POPS between Models #2 and #4. A PI(3,4)P2 hydration difference of ~7% was seen between Models #2 and #4. In addition, Na^+^ ions neutralize the membrane surface, when PI(3,4)P2 was present in a high concentration; however, a similar effect was not seen for POPS. This effect on surface charge or adherence of cations due to anionic lipid composition changes could also affect the dynamics of other solutes such as proteins or small molecules.

The results described above highlight a persistent problem: an over-simplification of the membrane environment in both in silico and in vitro studies of membrane proteins. This is commonplace even though the importance of a specific lipid environment for the membrane protein is recognized. For example, cholesterol, a lipid significantly modulating the structure of the lipid bilayer, is also known to affect a protein’s structure, dynamics, and function [54,55,56]. Likewise, polyunsaturated lipids regulate the behavior of G-protein coupled receptors [57,58]. As a single structural parameter, the membrane thickness is recognized as an essential factor affecting membrane proteins in multiple ways via the so-called hydrophobic mismatch mechanism [59,60,61]. Nevertheless, these three examples concern transmembrane proteins and structural properties of lipid bilayers, while the relation between properties of the water-membrane interface and peripheral, monotopic or bitopic membrane proteins remains elusive. A few studies have showed that changes in the behavior of the lipid headgroup, due to the membrane composition or the presence of divalent cations, might prevent the association of peripheral membrane proteins with the membrane [62,63].

In the context of OMM, the increased POPS levels have been linked to monoamine oxidase B (MOA-B) inactivation [13] and schizophrenia [14] decades ago. Likewise, the MAO-B activity has been linked to progressive brain diseases such as Alzheimer’s disease and Parkinson’s disease [16,64]. Accordingly, the increase of the POPS concentration at the OMM is potentially causing indirect or direct effects on the membrane protein’s structure and function. Firstly, the POPS-rich membrane could restrict the diffusion of monoamines (e.g., dopamine) by binding them more firmly onto the membrane surface. The anionic lipids are known to increase membrane adherence of amphipathic non-peptidic neurotransmitters in particular monoamines [6], e.g., dopamine, which likely accesses or enters its post-synaptic receptor via membrane-mediated pathway [65]. Secondly, it is also possible that increased POPS levels affect the MOA-B structure and dynamics adversely at the OMM, i.e., the monoamine entry via the membrane surface is hindered, or the subsequent catalysis is prevented due to a conformational shift. The opening of channels leading towards the catalytic center of MOA-A and -B occurs at the membrane surface [27,66,67], and loops controlling entrance to the channel are affected by the interactions with the nearby lipids [27]. Moreover, Cytochrome P, a protein of similar topology to MOA (single transmembrane helix and globular extra membrane catalytic domain), has been shown to be affected by lipid composition changes such as the addition of cholesterol [68] or charged lipids [69].

## 5. Conclusions

The molecular dynamics (MD) simulations of the five outer mitochondrial membrane (OMM; Figure 5) models indicate that the membrane lipid composition (MLC) has a relatively small effect on the membrane structural properties such as membrane thickness, area per lipid, and S_CD_ parameter. Importantly, the changes in the MLC had notable effects on the dynamical parameters of the models. Firstly, the simulations suggest on interesting behavior of ions at the membrane surface, which can be explained by the chemical structure of phosphatidylinositol. Secondly, there were marked changes at the water-membrane interface due to the MLC changes that are explained by the headgroup rotational data. Due to these apparent dynamic effects, it is recommended to probe the OMM-related phenomena such as small-molecule binding and permeation or protein dynamics using complex multi-component membrane models in the MD simulations that more accurately reflect the biochemical context (e.g., Model #2; Table 1; Figure 5).

## Figures and Tables

**Table 1:** Membrane lipid compositions of the outer mitochondrial membrane models.

Phospholipid Percentage	Model #1	Model #2	Model #3	Model #4	Model #5
POPC	59%	57%	50%	43%	57%
POPE	31%	31%	24%	17%	43%
POPS	0%	2%	20%	39%	0%
PI(3,4)P2	10%	10%	6%	1%	0%

**Table 2:** Area per lipid, membrane thickness, charge carried by bilayer, and surface charge density.

	Model #1	Model #2	Model #3	Model #4	Model #5
Area per lipid [nm^2^] ± 0.02	0.621	0.620	0.617	0.616	0.612
Total charge of lipids [e]	−312	−328	−332	−338	0
Surface charge density [e/nm^2^]	−0.320	−0.335	−0.345	−0.350	0
Total charge (lipids and Na^+^) [e]	−104	−104	−157	−213	18
Thickness [nm] ± 0.05	4.06	4.13	4.10	4.13	4.19

**Table 3:** Translational diffusion coefficients. An error estimate, which is the difference of the diffusion coefficients obtained from fits over the two halves of the fit interval, is given in parenthesis (). Not applicable (N/A).

	Model #1	Model #2	Model #3	Model #4	Model #5
POPC	8.4 (±0.7)	6.3 (±0.6)	7.2 (±0.7)	6.1 (±0.1)	8.4 (±0.7)
POPE	8.6 (±0.9)	6.2 (±0.7)	7.4 (±0.1)	5.6 (±0.7)	9.1 (±0.6)
POPS	N/A	3.5 (±1.4)	6.3 (±0.9)	2.9 (±2.9)	N/A
PI(3,4)P2	8.1 (±1.9)	4.8 (±3.2)	5.9 (±0.6)	5.1 (±0.3)	N/A

**Table 4:** Average number of water molecules in contact with the lipids via hydrogen bonding. Not applicable (N/A).

	Model #1	Model #2	Model #3	Model #4	Model #5
POPC	6.92	6.91	6.90	6.91	6.83
POPE	7.55	7.59	7.45	7.53	7.95
POPS	N/A	13.44	12.98	12.79	N/A
PI(3,4)P2	20.01	19.26	20.13	20.63	N/A

**Table 5:** Number of direct interactions between lipid molecules: H-bonds and charge pairs. Numbers given in [] are the predicted number based on the lipid concentration only and assuming no other specific interactions. The results marked with an asterisk (*) are calculated for Model #1 but are expected to be equal for Model #2. Not applicable (N/A).

Partner Lipid	Model #1	Model #2	Model #3	Model #4	Model #5
	H-bonds formed by POPE per POPE molecule				
POPC	0.48 [0.82]	0.41 [0.76]	0.38 [0.80]	0.32 [0.75]	0.49 [0.58]
POPE	0.45 [0.45]	0.45 [0.42]	0.32 [0.38]	0.24 [0.30]	0.53 [0.42]
POPS	N/A	0.04 [0.03]	0.59 [0.32]	1.14 [0.68]	N/A
PI(3,4)P2	0.48 [0.14]	0.44 [0.13]	0.31 [0.10]	0.05 [0.02]	N/A
All	1.41	1.34	1.60	1.75	1.02
	H-bonds formed by POPS per POPS molecule				
POPC	N/A	0.44 [0.76]	0.29 [0.79]	0.23 [0.65]	N/A
POPE	N/A	0.62 [0.42]	0.73 [0.38]	0.49 [0.26]	N/A
POPS	N/A	0.27 [0.03]	0.42 [0.31]	0.76 [0.59]	N/A
PI(3,4)P2	N/A	0.01 [0.13]	0.13 [0.09]	0.03 [0.01]	N/A
All	N/A	1.34	1.57	1.51	N/A
	H-bonds formed by PI(3,4)P2 per PI(3,4)P2 molecule				
POPC	0.17	0.17	0.12	0.16	N/A
POPE	1.49	1.37	1.36	1.08	N/A
POPS	N/A	0.00	0.45	1.08	N/A
PI(3,4)P2	1.86	1.86	1.86	1.98	N/A
All	3.52	3.40	3.79	4.3	N/A
	H-bonds formed by POPC per POPC molecule				
POPE	0.25 [0.21]	0.22 [0.19]	0.18 [0.14]	0.12 [0.11]	0.36
POPS	N/A	0.02 [0.02]	0.11 [0.12]	0.21 [0.22]	N/A
PI(3,4)P2	0.03 [0.07]	0.03 [0.06]	0.01 [0.02]	0.00 [0.00]	N/A
All	0.28	0.27	0.30	0.33	0.36
	Charge pairs formed by POPC per POPC molecule (or POPC per partner lipid)				
POPC	1.89	1.84	1.66	1.52	1.75
POPE	0.69 (1.33) *	0.69 (1.33) *	0.56 (1.15)	0.39 (0.69)	0.94 (1.25)
POPS	N/A *	N/A *	0.74 (1.88)	1.29 (1.42)	N/A
PI(3,4)P2	0.70 (4.14)	0.69 (3.94)	0.40 (3.55)	0.1 (4.25)	N/A
All	3.28 *	3.28 *	3.36	3.30	2.69

**Table 6:** Number of interactions between sodium ions and lipids.

	Model #1	Model #2	Model #3	Model #4	Model #5
Number of Na^+^ bound per lipid	0.27	0.29	0.22	0.16	0.02
Multiplicity	3.14	3.24	2.49	1.80	1.56
Number of Na^+^ bound per lipid—carbonyl groups	0.05	0.10	0.09	0.08	0.01

## Supplementary Materials

The following are available online at https://www.mdpi.com/article/10.3390/biom12020183/s1, Figure S1: The total energy of the simulated membrane model systems plotted against time, Figure S2: The temperature of the simulated membrane model systems plotted against time; Figure S3: The pressure of the simulated membrane model systems plotted against time. The NAMD input files needed for repeating or extending the simulations performed in this study and final configurations obtained after 300 ns simulation are containing in the following ZIP file (simulated_systems.zip).
