# Supplementary material for: Examining the Effect of Charged Lipids on Mitochondrial Outer Membrane Dynamics Using Atomistic Simulations
Source: Biomolecules. 2022 Jan 22;12(2):183. doi: 10.3390/biom12020183 (PMC8961577; doi:10.3390/biom12020183)
Supplement: Supplementary file 1 [file biomolecules-12-00183-s001.zip › Supplementary Materials/Supplementary Materials.pdf]

Supplementary Materials for

Examining the Effect of Charged Lipids on Mitochondrial Outer Membrane Dynamics Using Atomistic Simulations

Aline A. Oliveira [1,2,†] , Tomasz Róg [3,†,‡] , Albérico B. F. da Silva [2], Rommie E. Amaro [1], Mark S. Johnson [4,5] and Pekka A. Postila [1,4,5,6,7,8,*]

[1] Department of Chemistry and Biochemistry, University of California San Diego, San Diego, CA 92093-0340, USA; line_quimica@hotmail.com (A.A.O.); ramaro@ucsd.edu (R.E.A.)
[2] Instituto de Química de São Carlos, Universidade de São Paulo, CP 780, São Carlos 13560-970, SP, Brazil; alberico@iqsc.usp.br
[3] Department of Physics, University of Helsinki, P.O. Box 64, FI-00014 Helsinki, Finland; tomasz.rog@helsinki.fi
[4] Structural Bioinformatics Laboratory, Biochemistry, Faculty of Science and Engineering, Åbo Akademi University, 20520 Turku, Finland; mark.s.johnson@abo.fi
[5] InFLAMES Research Flagship Center, Åbo Akademi University
[6] Institute of Biomedicine, Kiinamyllynkatu 10, Integrative Physiology and Pharmacy, University of Turku, FI-20520 Turku, Finland
[7] Aurlide Ltd., Fi-21420 Lieto, Finland
[8] InFLAMES Research Flagship Center, University of Turku
* Correspondence: pekka.postila@utu.fi
† These authors contributed equally to this work.
‡ The author is deceased.

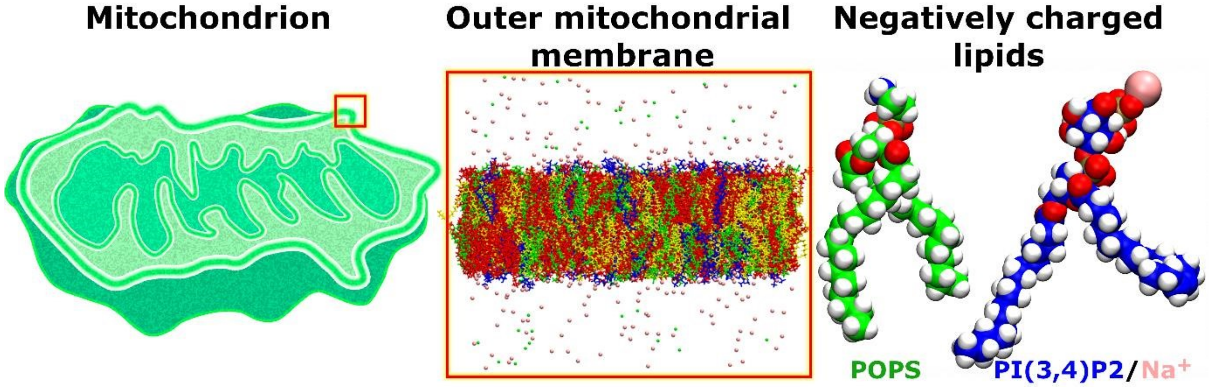

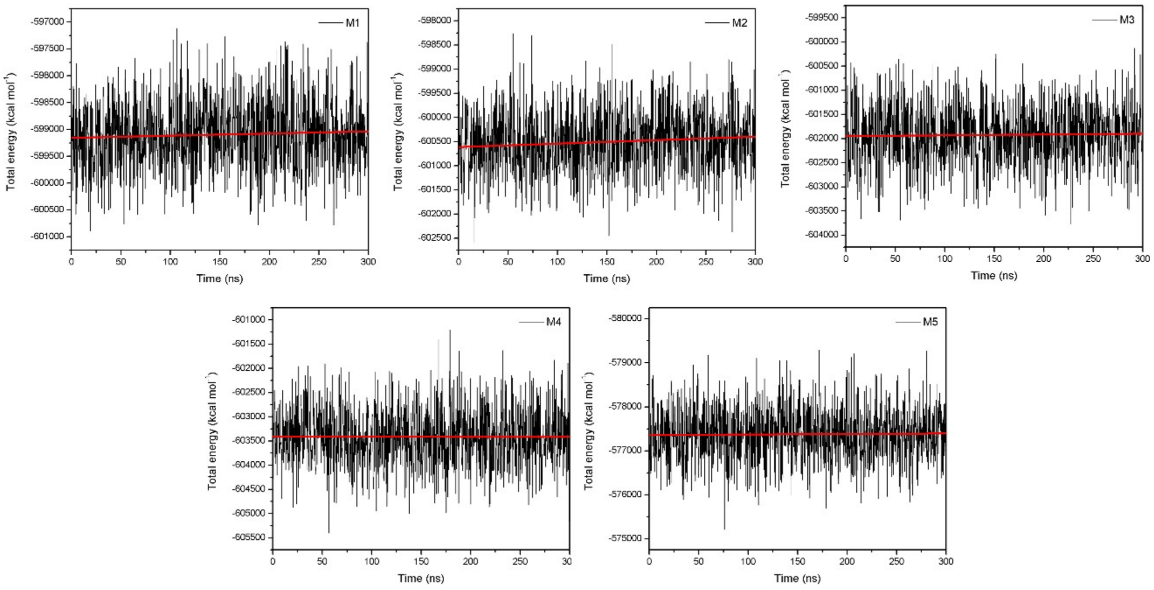

Figure S1. The total energy of the simulated membrane model systems plotted against time. The trend line is plotted with red color.

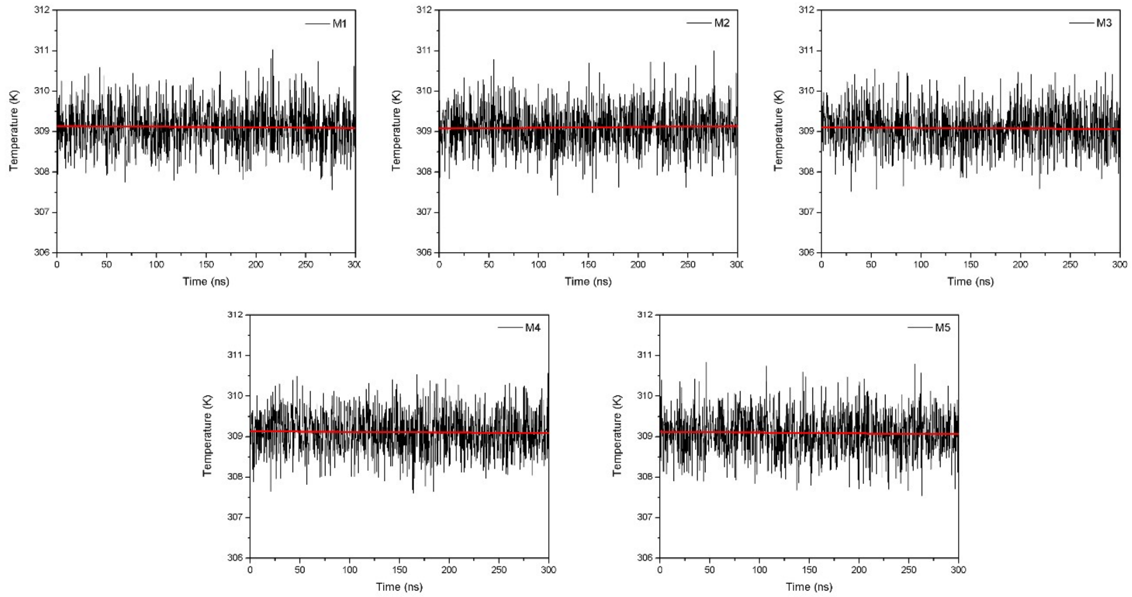

Figure S2. The temperature of the simulated membrane model systems plotted against time. The trend line is plotted with red color.

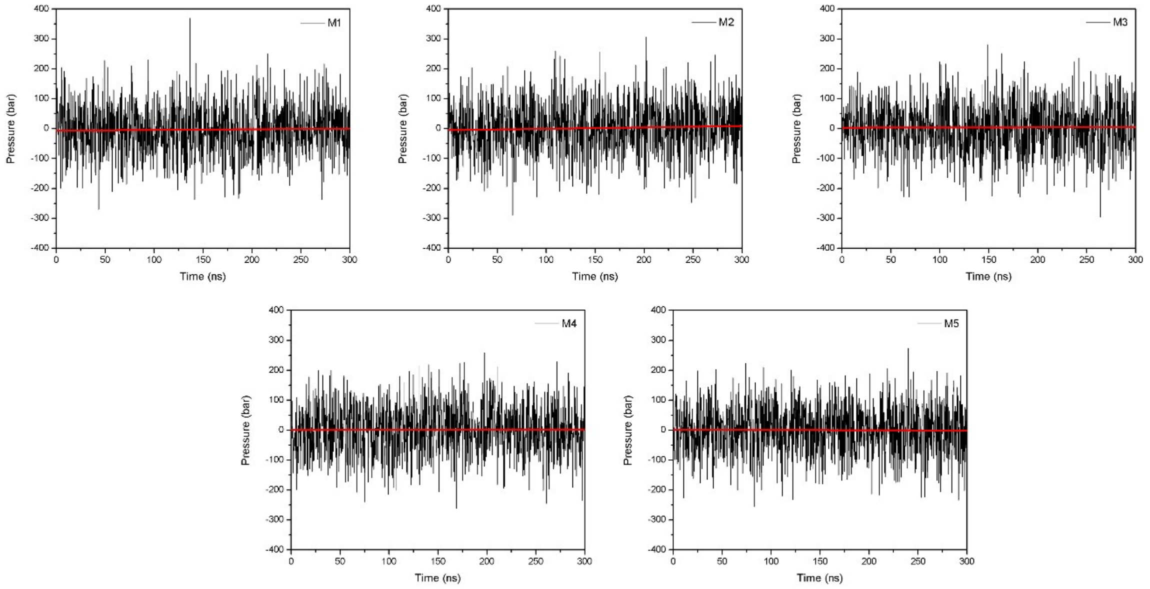

Figure S3. The pressure of the simulated membrane model systems plotted against time. The trend line is plotted with red color.

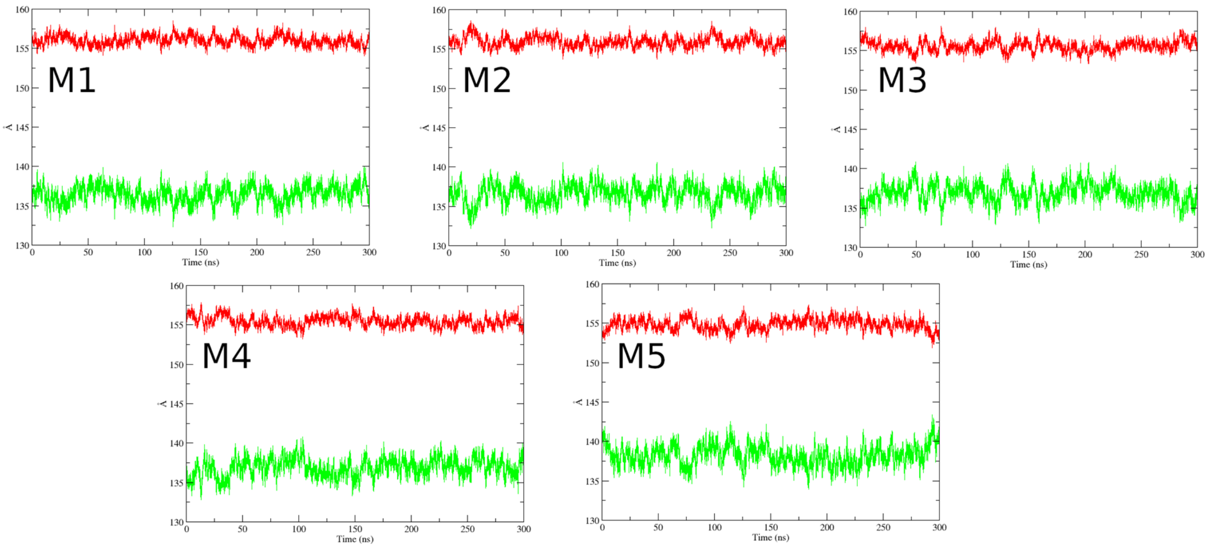

Figure S4. The box dimensions of the simulated membrane model systems plotted against time. The identical X- and Y- axis dimensions are shown by single red line, whereas the green line depicts the Z-axis. The average (0-300 ns) box dimensions (Å) for Models #1 (X or Y = 156.0; Z = 136.5), #2 (X or Y = 155.9; Z = 136.7), #3 (X or Y = 155.6; Z = 136.9), #4 (X or Y = 155.5; Z = 136.9), and #5 (X or Y = 154.8; Z = 138.2) were also calculated.

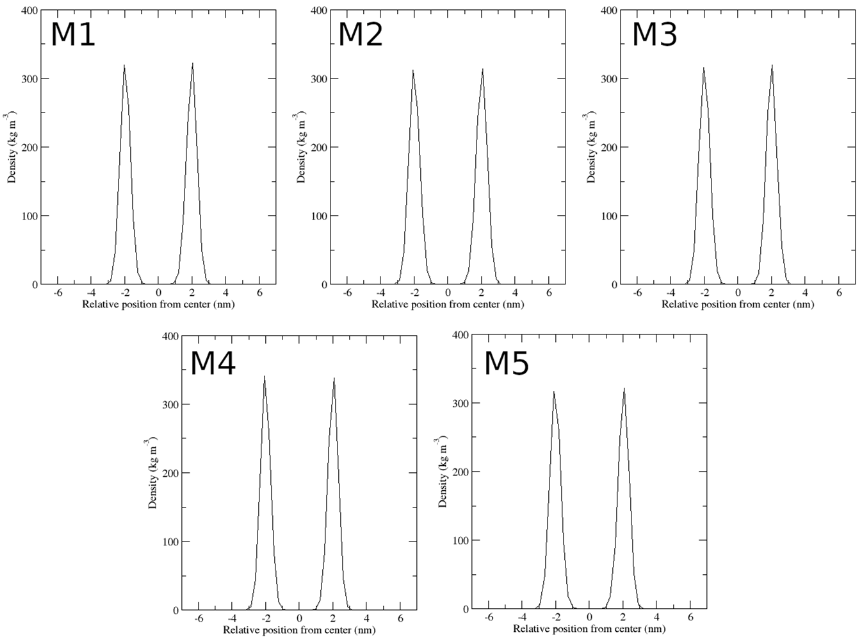

Figure S5. The partial density profile used in the membrane thickness calculation of the simulated membrane model systems. The lipid headgroup regions of the membrane leaflets are shown as black line that spans on both sides of the bilayer center (0).

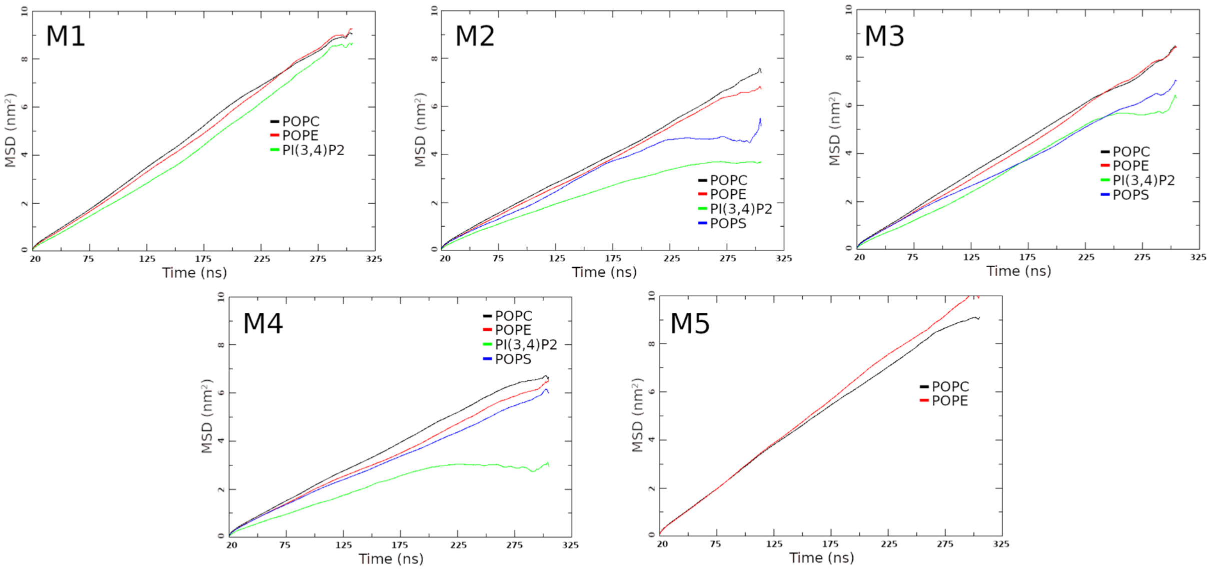

Figure S6. The mean squared displacement (MSD) of the center of mass of the lipids molecules in the bilayer plane of the simulated membrane model systems plotted against time.
